# Biliary and Pancreatic Safety of Incretin-Based Therapies in Obesity and Type 2 Diabetes: A Propensity-Matched Real-World Comparison of Tirzepatide, Semaglutide, and DPP-4 Inhibitors

**DOI:** 10.3390/jcm15208027

**Published:** 2026-10-16

**Authors:** Wissam Ghusn, Sasha-Jane Abi-Aad, Maria Daniela Hurtado

**Affiliations:** 1 Precision Medicine for Obesity Program, Division of Gastroenterology and Hepatology, Department of Medicine, Mayo Clinic, Rochester, MN 55905, USA; wissamghusn7@gmail.com; 2 Department of Internal Medicine, Saint Louis University, St. Louis, MO 63104, USA; sashajabiaad@gmail.com; 3 Division of Endocrinology, Diabetes, Metabolism, and Nutrition, Department of Medicine, Mayo Clinic, Jacksonville, FL 32224, USA

**Keywords:** biliary disease, GLP1-RA, semaglutide, tirzepatide, weight loss

## Abstract

To compare the 1-year risk of biliary/pancreatic adverse events among patients with obesity and type 2 diabetes treated with glucagon-like peptide-1 receptor agonists (GLP-1RAs) versus dipeptidyl peptidase-4 inhibitors (DPP-4i), and to directly compare tirzepatide with semaglutide. **Methods**: We conducted a retrospective propensity score-matched cohort study using the TriNetX Global Collaborative Network. Adults with obesity and type 2 diabetes without prior biliary or pancreatic disease were included. Two prespecified comparisons were performed: GLP-1RA (semaglutide or tirzepatide) versus DPP-4i, and tirzepatide versus semaglutide. The primary outcome was incident cholelithiasis at 1 year. Secondary outcomes included cholecystitis, cholangitis, acute pancreatitis, and cholecystectomy. Hazard ratios (HRs) were estimated using Cox proportional hazards models. **Results**: After matching, 281,283 patients were included in each GLP-1RA and DPP-4i cohort, and 242,876 patients in each tirzepatide and semaglutide cohort. Compared with DPP-4i, GLP-1RA therapy was associated with lower risks of cholelithiasis (1.02% vs. 1.16%; HR 0.92, 95%CI 0.87–0.96), cholecystitis (HR 0.88, 95%CI 0.79–0.98), cholangitis (HR 0.46, 95%CI 0.36–0.58), acute pancreatitis (HR 0.68, 95%CI 0.61–0.76), and cholecystectomy (HR 0.51, 95%CI 0.38–0.69). In the head-to-head comparison, semaglutide was associated with higher risks of cholelithiasis (HR 1.11, 95%CI 1.05–1.18) and cholecystitis (HR 1.22, 95%CI 1.10–1.37) compared with tirzepatide, while risks of pancreatitis, cholangitis, and cholecystectomy did not differ significantly. **Conclusions**: In this large real-world study, GLP-1RA therapy was associated with lower risks of biliary and pancreatic events compared with DPP-4i. Tirzepatide demonstrated a modestly more favorable biliary safety profile than semaglutide, supporting the overall hepatobiliary safety of contemporary GLP-1-based therapies.

## 1. Introduction

Obesity is a chronic, relapsing disease affecting over 40% of adults in the United States and is a major driver of type 2 diabetes, cardiovascular disease, and hepatobiliary morbidity [1,2]. Beyond its metabolic consequences, obesity is independently associated with gallstone formation, biliary dyskinesia, and pancreatitis through mechanisms including insulin resistance, altered bile acid metabolism, and chronic low-grade inflammation [3,4]. As obesity prevalence continues to rise globally, the long-term safety of therapies used to treat it, particularly with respect to biliary and pancreatic outcomes, has become an increasingly important clinical and public health concern.

Glucagon-like peptide-1 receptor agonists (GLP-1RAs) have transformed the management of obesity and type 2 diabetes, delivering substantial and durable improvements in glycemic control, weight loss, and cardiometabolic risk [5]. Their widespread adoption across endocrinology, primary care, and gastroenterology has been further accelerated by emerging evidence of cardiovascular and renal benefit [6,7]. As a result, millions of patients are now exposed to long-term GLP-1-based therapy, underscoring the importance of precisely defining their safety profile beyond metabolic outcomes.

Despite these benefits, concerns regarding biliary and pancreatic adverse events have persisted since early clinical trials and post-marketing surveillance reports [8]. Mechanistically, GLP-1 signaling may alter gallbladder motility, bile composition, and pancreatic exocrine function, raising biological plausibility for gallstone disease and pancreatitis [9,10,11,12]. However, the existing literature remains conflicted, and rigorous real-world comparisons have been limited by confounding, heterogeneous populations, and inadequate adjustment for obesity-related baseline risk.

GLP-1RAs belong to a broader pharmacological class known as incretin-based therapies, agents that harness the physiological effects of incretin hormones. Incretin hormones are gut-derived peptides released postprandially that augment insulin secretion and suppress glucagon in a glucose-dependent manner. Dipeptidyl peptidase-4 inhibitors (DPP-4i) represent a distinct but mechanistically related member of this class: rather than directly activating incretin receptors like GLP-1RAs, they prolong the half-life of endogenous GLP-1 and glucose-dependent insulinotropic polypeptide (GIP) by inhibiting the enzyme responsible for their degradation. Although DPP-4 inhibitors produce more modest weight-neutral glycemic effects compared to GLP-1RAs, they share a common incretin-mediated pathway and have been widely used as a guideline-recommended comparator in pharmacoepidemiologic research.

Prior studies have reported a significant increase in bile-duct and gallbladder disease with GLP-1RAs relative to DPP-4i [13]. However, the existing literature remains conflicted, and whether this risk extends to newer dual GIP/GLP-1 receptor agonists such as tirzepatide, and how it compares across the incretin class as a whole, remains insufficiently characterized.

Tirzepatide produces greater weight loss and metabolic improvement than earlier GLP-1RAs, effects that may differentially influence biliary physiology and gallstone risk [14]. Yet, to date, no large-scale real-world study has directly compared biliary and pancreatic safety outcomes between tirzepatide and semaglutide, the most widely prescribed GLP-1RA globally. This represents a critical evidence gap, particularly as clinicians increasingly choose between these agents for long-term therapy.

To address this unmet need, we conducted a large, propensity-matched, real-world cohort study evaluating the 1-year incidence of biliary and pancreatic events among adults with obesity and type 2 diabetes treated with tirzepatide, semaglutide, and DPP-4i. By leveraging a contemporary, nationally representative database and rigorously controlling for established biliary and pancreatic risk factors, this study aims to provide more definitive comparative safety data to inform clinical decision-making at a time of unprecedented GLP-1RA utilization.

## 2. Methods

### 2.1. Data Source

This retrospective cohort study was conducted using the TriNetX Global Collaborative Network, a federated research platform that provides access to de-identified electronic health record data from over 160 participating healthcare organizations. The database includes longitudinal information on demographics, diagnoses, procedures, medications, and laboratory values collected during routine clinical care. All analyses were performed within the TriNetX analytics environment, which is compliant with HIPAA regulations and qualifies as non-human subjects’ research.

### 2.2. Study Population and Eligibility Criteria

Adult patients aged 18 years or older with obesity and type 2 diabetes were eligible for inclusion. Obesity was defined as a body mass index (BMI) ≥ 30 kg/m^2^, and diabetes was identified using ICD-10-CM codes E08-E13. To ensure evaluation of incident biliary and pancreatic outcomes, patients were excluded if they had any documented history prior to cohort entry of cholelithiasis, cholecystitis, cholangitis, other biliary tract disease, acute pancreatitis, prior cholecystectomy or cholecystostomy, primary biliary cholangitis, primary sclerosing cholangitis, or biliary or gallbladder malignancy. Patients with prior exposure to the comparator drug class were also excluded to prevent treatment contamination.

### 2.3. Exposure Definitions and Cohort Construction

Two prespecified comparative analyses were performed. In the first analysis, patients initiating GLP-1RA therapy were compared with those initiating DPP-4i. The GLP-1RA cohort included patients with at least two prescriptions for semaglutide or tirzepatide between 1 January 2020 and 1 November 2025, with no prior DPP-4i exposure. The DPP-4i cohort included patients with at least two prescriptions for sitagliptin or linagliptin during the same period, which are the most commonly prescribed DPP-4i medications, with no prior GLP-1RA use. Requiring at least two prescriptions also increases the likelihood that therapy was approved by insurance and initiated, reflecting sustained access to treatment rather than denied, delayed, or discontinued prescriptions. In the second analysis, patients treated with tirzepatide were directly compared with those treated with semaglutide. Each cohort required at least two prescriptions of the respective medication and no prior exposure to the alternative agent. For all cohorts, the index date was defined as the date of first qualifying prescription after all eligibility criteria were satisfied.

### 2.4. Follow-Up

Patients were followed from one day after the index date for up to 365 days or until the last recorded clinical encounter, whichever occurred first. Outcomes occurring on or before the index date were not counted, minimizing immortal time bias and reverse causation.

### 2.5. Outcomes

The primary outcome was incident cholelithiasis, defined using ICD-10-CM code K80. Secondary outcomes included cholecystitis (K81), cholangitis (K83.0), acute pancreatitis (K85), and cholecystectomy or cholecystostomy identified using validated CPT, SNOMED, and ICD-9 procedure codes. ICD-9 procedure codes were included to account for historical and international coding variations within the TriNetX Global Collaborative Network. All outcomes were assessed as first incident events during the follow-up period.

### 2.6. Covariates and Propensity Score Matching

To control for confounding, 1:1 propensity score matching without replacement was performed separately for each comparison using a greedy nearest-neighbor algorithm. Covariates were selected a priori based on established associations with biliary and pancreatic disease and included age, sex, race, ethnicity, BMI, alcohol use disorder, hypertriglyceridemia, estrogen exposure, and markers of diabetes severity, including hemoglobin A1c and insulin use. Covariate balance before and after matching was assessed using standardized mean differences, with values < 0.1 indicating adequate balance.

### 2.7. Statistical Analysis

Baseline characteristics were summarized using descriptive statistics before and after propensity score matching to evaluate the distribution and balance of demographic and clinical variables between cohorts. All outcome analyses were conducted within the propensity-matched populations. Time-to-event associations between treatment exposure and each outcome were evaluated using Cox proportional hazards regression models, from which hazard ratios and corresponding 95% confidence intervals were derived. To account for differences in available follow-up time between patients and treatment cohorts, patients were censored at the time of their last recorded clinical encounter, such that each patient contributed observed time at risk until the occurrence of an outcome or censoring. The proportional hazards assumption was assessed using standard diagnostics within the analytic platform.

## 3. Results

### 3.1. Study Population

In the GLP-1RA versus DPP-4i analysis, the unmatched cohorts included 900,357 GLP-1RA users and 418,205 DPP-4i users. Before matching, GLP-1RA users were younger (mean age 52.8 ± 13.9 vs. 65.2 ± 13.4 years), more frequently female (63.9% vs. 47.1%), and had higher mean BMI (37.8 ± 7.7 vs. 31.2 ± 7.3 kg/m^2^). After 1:1 propensity score matching, 281,283 patients remained in each group. Most measured covariates demonstrated improved balance after matching; however, residual imbalance remained for BMI (36.1 ± 7.36 vs. 32.0 ± 7.32 kg/m^2^; SMD 0.56) and HbA1c (7.53 ± 1.88% vs. 8.10 ± 1.84%; SMD 0.25) between the GLP-1RA and DPP-4i cohorts. Mean follow-up after matching was 314.5 ± 95.2 versus 329.8 ± 89.7 days, respectively (Table 1). For the tirzepatide versus semaglutide comparison, the unmatched cohorts included 242,876 tirzepatide users and 690,147 semaglutide users. After propensity score matching, 242,876 patients were retained in each cohort with well-balanced baseline characteristics. Mean follow-up duration after matching was 273.8 ± 115.0 days for tirzepatide and 331.6 ± 114.9 days for semaglutide (Table 2).

### 3.2. Incidence of Cholelithiasis

**Figure 1:**
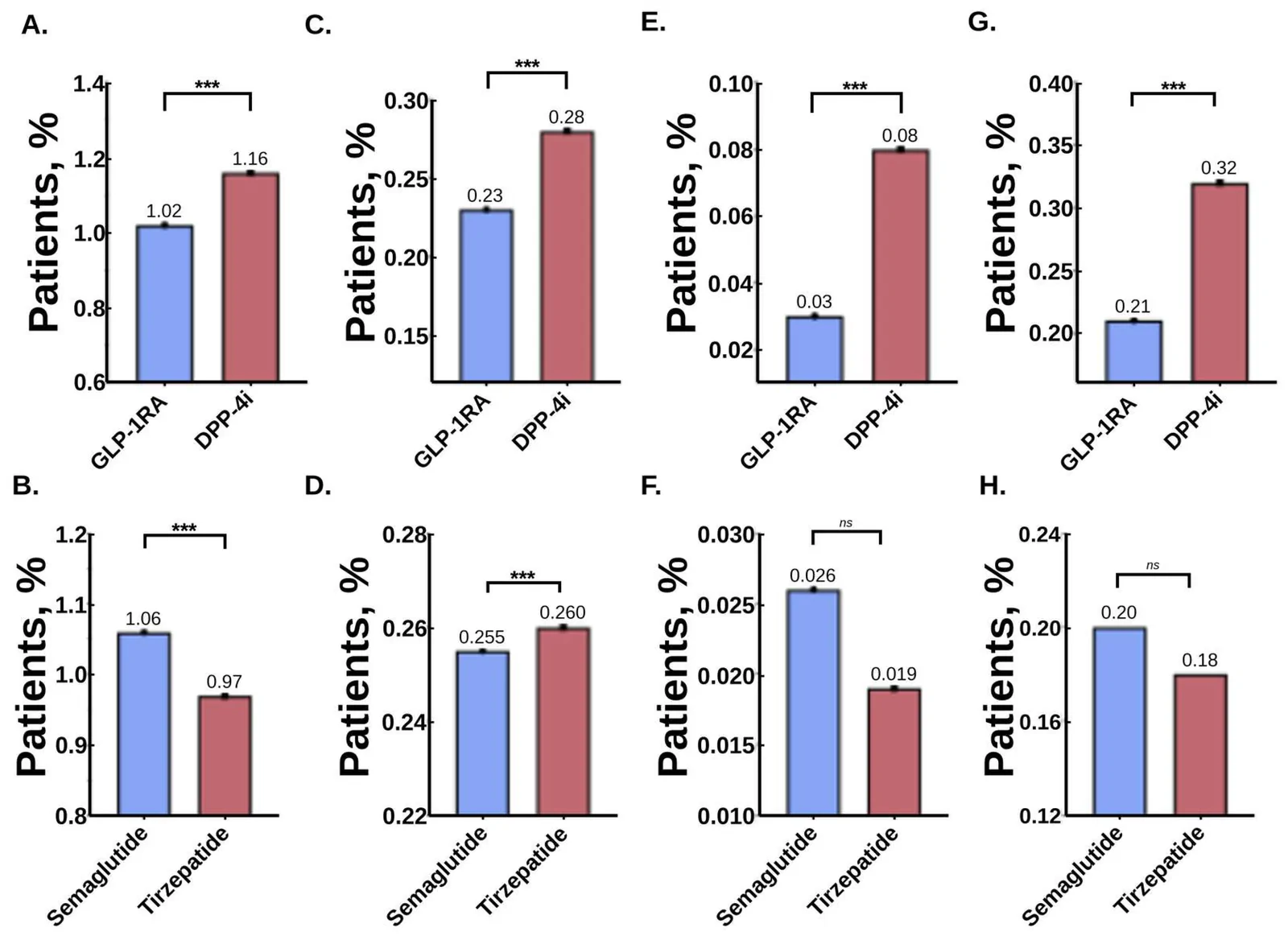
Comparative Risk of Biliary and Pancreatic Events with Incretin-Based Therapies. Panels (**A**–**H**) show the 1-year incidence of (**A**,**B**) cholelithiasis, (**C**,**D**) cholecystitis, (**E**,**F**) cholangitis, and (**G**,**H**) acute pancreatitis for GLP-1 receptor agonists vs. DPP-4 inhibitors and semaglutide vs. tirzepatide, respectively.***: *p* < 0.01.

**Figure 2:**
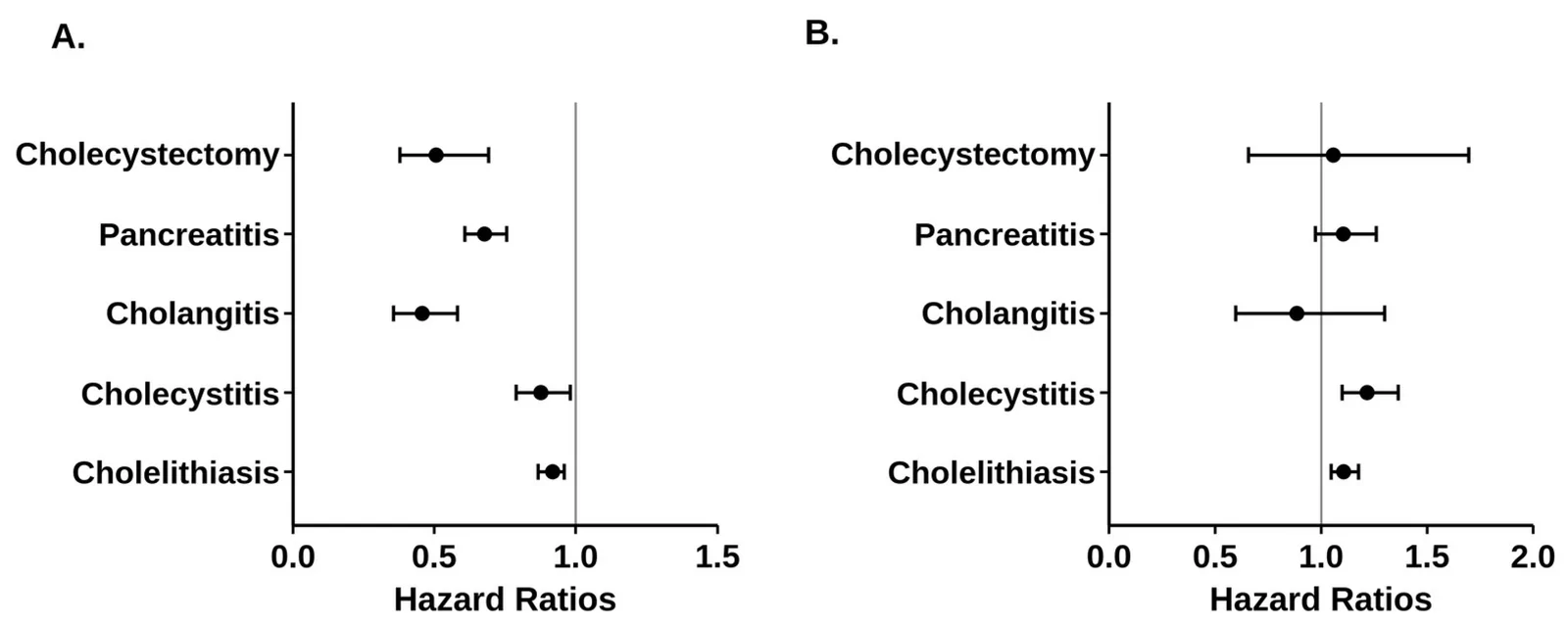
Comparative biliary and pancreatic events with GLP-1 receptor agonists compared to DPP-4 inhibitors (**A**) and semaglutide compared to tirzepatide (**B**). HR < 1 in Panel A indicates a lower hazard with GLP-1RA therapy, whereas HR > 1 in Panel (**B**) indicates a higher hazard with semaglutide relative to tirzepatide.

Over 12 months of follow-up in the propensity-matched GLP-1RA versus DPP-4 inhibitor cohorts, cholelithiasis occurred in 2863 patients (1.02%) receiving GLP-1RA therapy and 3275 patients (1.16%) receiving DPP-4i (Figure 1A). GLP-1RA therapy was associated with a significantly lower risk of cholelithiasis compared with DPP-4 inhibitors (HR 0.92, 95% CI 0.87–0.96; Figure 2A).

In the matched tirzepatide versus semaglutide analysis, cholelithiasis occurred in 2345 patients (0.97%) treated with tirzepatide and 2564 patients (1.06%) treated with semaglutide during follow-up (Figure 1B). Semaglutide use was associated with a higher risk of cholelithiasis compared with tirzepatide (HR 1.11, 95% CI 1.05–1.18; Figure 2B).

### 3.3. Incidence of Cholecystitis

Cholecystitis occurred in 653 patients (0.23%) in the GLP-1RA cohort and 782 patients (0.28%) in the DPP-4i cohort over 12 months of follow-up (Figure 1C). GLP-1RA therapy was associated with a significantly lower risk of cholecystitis compared with DPP-4i (HR 0.88, 95% CI 0.79–0.98; Figure 2A).

In the tirzepatide versus semaglutide comparison, cholecystitis occurred in 620 patients (0.255%) receiving tirzepatide and 631 patients (0.26%) receiving semaglutide (Figure 1D). Semaglutide was associated with a significantly higher risk of cholecystitis compared with tirzepatide (HR 1.22, 95% CI 1.10–1.37; Figure 2B).

### 3.4. Incidence of Cholangitis

Cholangitis was rare in both treatment groups but occurred less frequently among GLP-1RA users, affecting 92 patients (0.03%) compared with 211 patients (0.08%) treated with DPP-4i (Figure 1E). GLP-1RA therapy was associated with a markedly lower risk of cholangitis (HR 0.46, 95% CI 0.36–0.58; Figure 2A). In the tirzepatide versus semaglutide cohorts, cholangitis occurred in 45 patients (0.019%) treated with tirzepatide and 63 patients (0.026%) treated with semaglutide (Figure 1F). No statistically significant difference was observed between groups (HR 0.89, 95% CI 0.60–1.30; Figure 2B).

### 3.5. Incidence of Acute Pancreatitis

Acute pancreatitis occurred in 590 patients (0.21%) receiving GLP-1RA therapy and 907 patients (0.32%) receiving DPP-4i during follow-up (Figure 1G). GLP-1RA use was associated with a significantly lower risk of acute pancreatitis compared with DPP-4i therapy (HR 0.68, 95% CI 0.61–0.76; Figure 2A).

In the tirzepatide versus semaglutide analysis, acute pancreatitis occurred in 447 patients (0.18%) treated with tirzepatide and 496 patients (0.20%) treated with semaglutide during follow-up, with no significant difference in risk between groups (HR 1.11, 95% CI 0.98–1.26; Figure 1H).

### 3.6. Incidence of Cholecystectomy and Biliary Procedures

Cholecystectomy or related biliary procedures occurred in 64 patients (0.02%) in the GLP-1RA cohort and 132 patients (0.05%) in the DPP-4i cohort. GLP-1RA therapy was associated with a significantly lower risk of cholecystectomy compared with DPP-4i therapy (HR 0.51, 95% CI 0.38–0.69; Figure 2A). In the tirzepatide versus semaglutide comparison, cholecystectomy was uncommon, occurring in 32 tirzepatide users (0.013%) and 38 semaglutide users (0.016%). No statistically significant difference was observed between treatment groups (HR 1.06, 95% CI 0.66–1.70; Figure 2B).

## 4. Discussion

In this large, propensity-matched real-world analysis of adults with obesity and type 2 diabetes, we demonstrated that GLP-1RA therapy was associated with significantly lower risks of biliary and pancreatic events compared with DPP-4i over one year of follow-up. Specifically, GLP-1RA use was associated with reduced hazards of cholelithiasis, cholecystitis, cholangitis, acute pancreatitis, and cholecystectomy. In a head-to-head comparison within the GLP-1RA class, tirzepatide demonstrated a consistently lower risk of cholelithiasis and cholecystitis compared with semaglutide, while risks of pancreatitis, cholangitis, and biliary procedures were low and did not differ significantly between agents. Together, these findings provide robust, contemporary comparative safety data at a time of unprecedented GLP-1RA utilization and directly address persistent concerns regarding hepatobiliary and pancreatic adverse events.

These results are clinically important for several reasons. First, obesity and diabetes independently increase the risk of gallstone disease and pancreatitis, making it essential to distinguish medication-associated risk from underlying disease burden [15]. By leveraging a large real-world database and propensity score matching across established biliary and pancreatic risk factors, our study provides comparative estimates of biliary and pancreatic outcomes among patients receiving incretin-based therapies. However, residual imbalance in baseline BMI and HbA1c remained after matching in the GLP-1RA versus DPP-4i comparison and should be considered when interpreting these associations. Second, the observed reduction in biliary events with GLP-1RA therapy compared with DPP-4i challenges the prevailing narrative that GLP-1RAs inherently increase gallbladder risk and suggests that, in real-world populations, these agents may be comparatively safer than commonly used alternative glucose-lowering therapies [16].

Our findings help reconcile inconsistencies in the existing literature. Early randomized trials and meta-analyses reported modest increases in gallbladder-related events among GLP-1RA users, particularly in the context of rapid weight loss [17]. In addition, observational studies have yielded conflicting results, frequently limited by residual confounding, heterogeneous populations, or inclusion of patients with pre-existing biliary disease [16,18]. Recent real-world evidence has also demonstrated differing findings regarding pancreatic safety. Faour et al. reported different associations between GLP-1RA initiation and acute pancreatitis in a large propensity-matched cohort, highlighting the potential influence of differences in study population, comparator selection, baseline risk, and analytic design. Similarly, Conforti et al. reported modest increases in pancreatic enzymes without clinically suspected pancreatitis during six months of semaglutide treatment in a smaller real-world cohort, providing complementary evidence regarding pancreatic safety in routine clinical practice [19]. Other prior evidence suggests that gallbladder risk with GLP-1RAs may vary over time. Faillie et al. reported an increased risk of biliary disease during the first 180 days of treatment, likely related to rapid weight loss and cholesterol supersaturation of bile [13], whereas a large meta-analysis by He et al. found that risks became more apparent with treatment durations exceeding 26 weeks [11]. Together, these findings suggest that early biliary events may be driven by rapid weight loss, while longer-term risk may reflect cumulative effects of sustained weight reduction and gallbladder hypomotility. Notably, our 12-month analysis did not demonstrate an increased biliary risk with GLP-1RA therapy compared with DPP-4i.

The lower risk of biliary events observed with GLP-1RA therapy compared with DPP-4i may reflect several mechanisms. GLP-1RAs improve insulin sensitivity [20], reduce hypertriglyceridemia [21], and exert anti-inflammatory effects [22], all of which may mitigate biliary and pancreatic risk. Additionally, while rapid weight loss is known to promote gallstone formation, sustained metabolic improvement and gradual weight reduction, more characteristic of real-world GLP-1RA use [21], may offset this risk over time. Interestingly, the particularly strong association observed for cholangitis and cholecystectomy suggests that GLP-1RA therapy may reduce progression from asymptomatic gallstones to clinically significant biliary disease.

An additional consideration is that DPP-4i themselves have been linked to gallbladder and biliary disease in some studies [12,23]. Therefore, the lower biliary event rates observed with GLP-1RAs in our study should be interpreted as comparative safety relative to DPP-4i rather than evidence of complete absence of biliary risk. This may partly explain the discrepancy between our findings and prior placebo-controlled studies that reported increased gallbladder events among GLP-1RA users.

Within the GLP−1RA class, the observed differences between tirzepatide and semaglutide warrant consideration. Tirzepatide’s dual GIP/GLP-1 mechanism produces greater metabolic improvement and weight loss than semaglutide [8], yet was associated with lower hazards of cholelithiasis and cholecystitis. This finding argues against a simplistic weight-loss-driven explanation for gallbladder risk and raises the possibility that GIP signaling, differential effects on gallbladder motility, or variations in prescribing patterns and dose escalation may influence biliary outcomes [24]. However, longitudinal weight trajectories and detailed dosing or titration patterns were not available in our analysis, precluding assessment of whether differences in the rate or magnitude of weight loss contributed to these findings. Accordingly, the potential role of GIP signaling or other agent-specific physiological effects remains speculative. Importantly, absolute event rates were low for both agents, and no significant differences were observed for pancreatitis or biliary procedures, supporting the overall biliary and pancreatic safety of contemporary incretin-based therapies.

This study has limitations inherent to retrospective analyses of electronic health record data. Residual confounding from unmeasured factors, such as dietary patterns, degree and tempo of weight loss, or medication adherence, cannot be fully excluded. Although requiring at least two prescriptions increased the likelihood of treatment initiation, continuous medication exposure and adherence could not be confirmed. Outcome misclassification is possible, although validated diagnostic and procedural codes were used. Follow-up was limited to one year, and longer-term biliary risk beyond this period remains uncertain. However, available evidence suggests that most GLP-1RA-associated biliary events occur within the first year of therapy initiation [13]. In addition, follow-up duration differed between treatment groups, with semaglutide users having longer mean follow-up than tirzepatide users after matching. Our use of Cox proportional hazards models accounts for variable observed follow-up through time-to-event analysis and censoring at the last recorded clinical encounter; however, residual differential ascertainment bias cannot be completely excluded. Sensitivity analyses requiring a fixed duration of continuous healthcare coverage or observation could not be reliably performed within the TriNetX analytic environment. Nevertheless, the large sample size, active comparator design, exclusion of baseline biliary disease, and consistent findings across multiple outcomes strengthen the validity and clinical relevance of our results.

## 5. Conclusions

In a large real-world population of adults with obesity and type 2 diabetes, GLP-1RA therapy was associated with lower observed risks of biliary and pancreatic events compared with DPP-4i, while tirzepatide demonstrated a modestly more favorable biliary safety profile than semaglutide. These findings provide contemporary comparative safety data for incretin-based therapies; however, residual differences in baseline BMI and HbA1c and the inability to account for the magnitude and rate of treatment-associated weight loss warrant cautious interpretation of the observed associations. Further studies incorporating longitudinal measures of body weight are needed to confirm these findings.

## Figures and Tables

**Table 1:** Baseline characteristics of GLP-1RA vs. DPP-4i cohorts before and after propensity score matching.

Characteristics	Before Matching		After Matching		
	GLP-1RA (*n* = 900,357)	DPP-4i (*n* = 418, 205)	GLP-1RA (*n* = 281,283)	DPP-4i (*n* = 281,283)	SMD
Age, mean ± SD in years	52.8 ± 13.9	65.2 ± 13.4	61.9 ± 11.9	61.6 ± 13.1	0.024
Sex, *n* (%)					
Female	575,414 (63.91)	197,076 (47.12)	137,494 (48.89)	141,506 (50.31)	0.029
Male	324,943 (36.09)	221,129 (52.88)	143,789 (51.812)	139,777 (49.69)	0.029
Race, *n* (%)					
White	623,787 (69.28)	172,598 (41.27)	160,102 (56.91)	156,176 (55.52)	0.028
Black	150,142 (16.67)	54,091 (12.93)	49,208 (17.49)	47,124 (16.75)	0.020
Asian	26,323 (2.92)	39,357 (9.41)	18,263 (6.49)	18,192 (6.47)	0.001
American Indian	4866 (0.54)	1386 (0.33)	1305 (0.46)	1263 (0.45)	0.002
Native Hawaiian	5230 (0.58)	2109 (0.50)	1937 (0.69)	1871 (0.66)	0.003
Ethnicity, *n* (%)					
Hispanic or Latino	242,401 (26.92)	196,266 (46.93)	91,316 (32.5)	99,155 (35.25)	0.059
Non-Hispanic	657,956 (73.07)	221,939 (53.07)	189,967 (67.54)	182,128 (64.75)	0.059
BMI, mean ± SD	37.8 ± 7.67	31.2 ± 7.3	36.1 ± 7.36	32 ± 7.32	0.56
Biliary/pancreatic disease risk factors					
Alcohol use disorder, *n* (%)	13,505 (1.5)	6273 (1.5)	3938 (1.4)	4219 (1.5)	0.008
Hypertriglyceridemia, *n* (%)	20,181 (2.24)	6057 (1.44)	5900 (2.10)	5192 (1.85)	0.018
HbA1c, mean ± SD	7.08 ± 1.88	7.96 ± 1.83	7.53 ± 1.88	8.1 ± 1.84	0.25
Insulin use, *n* (%)	141,503 (15.71)	111,151 (26.58)	80,965 (28.74)	71,467 (25.40)	0.076
Estrogen use, *n* (%)	4899 (0.54)	2503 (0.60)	1850 (0.66)	1941 (0.69)	0.002
Follow-up, mean ± SD in days	314.43 ± 95.46	320.77 ± 99.16	314.50 ± 95.15	329.83 ± 89.68	NA

SMD: Standardized mean difference; SMD < 0.1 indicates adequate covariate balance/matching.

**Table 2:** Baseline characteristics of tirzepatide vs. semaglutide (B) cohorts before and after propensity score matching.

Characteristics	Before Matching		After Matching		
	Tirzepatide (*n* = 242,876)	Semaglutide (*n* = 690,147)	Tirzepatide (*n* = 242,876)	Semaglutide (*n* = 242,876)	SMD
Age, mean ± SD in years	52.8 ± 13.7	54.3 ± 14.0	52.8 ± 13.7	52.8 ± 13.7	0.001
Sex, *n* (%)					
Female	152,423 (62.8%)	428,989 (62.2%)	152,423 (62.8%)	152,406 (62.8%)	<0.001
Male	90,453 (37.2%)	261,158 (37.8%)	90,453 (37.2%)	90,470 (37.2%)	<0.001
White	178,350 (73.4%)	458,389 (66.4%)	178,350 (73.4%)	178,382 (73.4%)	<0.001
Black	34,150 (14.1%)	122,380 (17.7%)	34,150 (14.1%)	34,243 (14.1%)	0.001
Asian	5916 (2.4%)	25,739 (3.7%)	5916 (2.4%)	5888 (2.4%)	0.001
American Indian	1147 (0.5%)	3874 (0.6%)	1147 (0.5%)	1045 (0.4%)	0.006
Native Hawaiian	1037 (0.4%)	4865 (0.7%)	1037 (0.4%)	999 (0.4%)	0.002
Ethnicity, *n* (%)					
Hispanic or Latino	178,946 (73.7%)	503,026 (72.9%)	178,946 (73.7%)	179,063 (73.7%)	0.001
Non-Hispanic	63,930 (26.3%)	187,121 (27.1%)	63,930 (26.3%)	63,813 (26.3%)	0.001
BMI, mean ± SD	37.8 ± 7.5	37.2 ± 7.7	37.8 ± 7.5	37.5 ± 7.7	0.039
Biliary/pancreatic disease risk factors					
Alcohol use disorder, *n* (%)	1237 (0.5%)	3745 (0.5%)	1237 (0.5%)	1010 (0.4%)	0.014
Hypertriglyceridemia, *n* (%)	5338 (2.2%)	16,917 (2.5%)	5338 (2.2%)	5039 (2.1%)	0.009
HbA1c, mean ± SD	7.0 ± 1.8	7.4 ± 1.9	7.0 ± 1.8	7.3 ± 1.9	0.157
Insulin use, *n* (%)	36,329 (15.0%)	136,012 (19.7%)	36,329 (15.0%)	36,298 (14.9%)	<0.001
Estrogen use, *n* (%)	7665 (3.2%)	18,271 (2.6%)	7665 (3.2%)	7457 (3.1%)	0.005
Follow-up, median ± SD in days	273.77 ± 114.99	331.99 ± 79.07	273.77 ± 114.99	331.65 ± 114.99	NA

SMD: Standardized mean difference; SMD < 0.1 indicates adequate covariate balance/matching.

## Data Availability

The data supporting the findings of this study were obtained from the TriNetX Global Collaborative Network. Restrictions apply to the availability of these data, which are not publicly available due to the TriNetX data use agreement.
